# Hydronephrosis Caused by Polyglycolic Acid Spacer Placement Prior to Carbon Ion Radiotherapy for Local Recurrence of Rectal Cancer: Case Report

**DOI:** 10.70352/scrj.cr.25-0395

**Published:** 2025-10-11

**Authors:** Katsuya Ueda, Kenji Matsuda, Hirotoshi Takiyama, Yasuyuki Mitani, Hiromitsu Iwamoto, Yuki Nakamura, Norio Takemoto, Takahiko Hyo, Kazuki Shimomura, Manabu Kawai

**Affiliations:** 1 Second Department of Surgery, School of Medicine, Wakayama Medical University, Wakayama, Wakayama, Japan; 2 QST Hospital, National Institutes for Quantum Science and Technology, Chiba, Chiba, Japan

**Keywords:** carbon ion radiotherapy, locally recurrent rectal cancer, polyglycolic acid spacer, hydronephrosis, laparoscopic surgery

## Abstract

**INTRODUCTION:**

Carbon-ion radiotherapy (CIRT) for pelvic recurrent rectal cancer has recently attracted attention due to its excellent therapeutic outcomes. A spacer is often inserted before CIRT to ensure a certain distance between the recurrent lesion and the adjacent intestine. We report a case of hydronephrosis due to ureteral stenosis after laparoscopic insertion of a polyglycolic acid (PGA) spacer.

**CASE PRESENTATION:**

A 58-year-old man underwent laparoscopic abdominoperineal resection and right lateral lymph node dissection after neoadjuvant chemoradiotherapy. He had been free of recurrence for 4 years. PET-CT 4.5 years after surgery revealed a 9-mm lymph node enlargement with an SUVmax = 2.88 in the left lateral region. Laparoscopic left lateral lymph node dissection was performed due to suspicion of recurrence, but removal was difficult due to severe fibrosis after previous radiation therapy. Definitive diagnosis of recurrence had not been made, so the patient was observed without treatment, but PET-CT 7 years after the initial surgery showed that the lymph node had enlarged to 25 mm with uptake of SUVmax = 8.06. Recurrence was strongly suspected, so we planned CIRT, and PGA spacer insertion was performed laparoscopically in advance. CT 3 days after insertion of the PGA spacer revealed hydronephrosis due to ureteral stenosis, which was thought to be caused by compression from the PGA spacer. Follow-up CT taken 7 days after surgery showed no improvement, so a ureteral stent was placed. Ureteral stenosis has persisted, so the ureteral stent has been replaced every 3 months. Regarding the recurrent lesions after CIRT, a tendency for them to shrink was observed on CT taken 1.5 years after the procedure.

**CONCLUSIONS:**

In this case, a PGA spacer was inserted laparoscopically and hydronephrosis occurred early due to ureteral stenosis. Although the ureteral stenosis in the early postoperative period was thought to be caused by the PGA spacer, the persistence of ureteral stenosis was thought to be a late adverse event of CIRT. In cases where the ureter is exposed, such as after lateral lymph node dissection with neoadjuvant chemoradiotherapy, prophylactic ureteral stenting before inserting a PGA spacer may be considered.

## Abbreviations

SUV
: standardized uptake value
UICC
: union for international cancer control

## INTRODUCTION

Local recurrence of advanced rectal cancer can be largely controlled by total mesorectal excision and neoadjuvant chemoradiotherapy, but local recurrence still occurs in approximately 5%–8% of cases.^1,2)^

Salvage surgery is performed for local recurrence if possible, but there are often cases in which reoperation is difficult. In such cases, radiation therapy may be an option, and carbon ion radiotherapy (CIRT) has recently attracted attention for its high local control rate and 5-year survival rate.^3)^ CIRT allows selective irradiation of tumors with high doses due to the Bragg peak, while minimizing exposure to surrounding organs.^4)^

CIRT has few side effects, but exposure of the small intestine to high doses of radiation can be problematic due to the development of perforations and ulcers, so the CIRT dose needs to be reduced near the digestive tract.^5)^ Spacers have been used to create a gap between the malignant tumor and the nearby digestive tract, thereby reducing the radiation dose to the digestive tract while delivering full dose to the recurrent tumor.^6)^ Recently, bio-absorbable polyglycolic acid (PGA) spacers that degrade via hydrolysis have been used in CIRT for the treatment of malignant pelvic tumors in Japan.^7)^ Known complications of PGA insertion include ileus and infection,^8,9)^ but there have been no reports of hydronephrosis. Here, we report a case of hydronephrosis that occurred after laparoscopic PGA spacer insertion.

## CASE PRESENTATION

The patient was a 58-year-old man whose chief complaint was bloody stool. Colonoscopy revealed rectal cancer. He had a medical history of appendicitis surgery, hypertension, and a history of contrast media allergy. His height, weight, and body mass index were 171.8 cm, 88 kg, and 29.8, respectively.

At the initial visit, CT and MRI revealed 8 mm of lymph node enlargement in the right lateral region, suggesting metastasis. After neoadjuvant chemoradiotherapy, laparoscopic abdominoperineal resection and right lateral lymph node dissection were performed, and the patient was diagnosed with ypT3N0M0: ypStage II a (UICC TNM classification, 8th edition). Genetic testing showed RAS mutant, BRAF wild, and microsatellite stable. The patient received oral fluoropyrimidine monotherapy for 6 months as postoperative adjuvant chemotherapy, and there was no recurrence for 4 years after surgery.

**Fig. 1:**
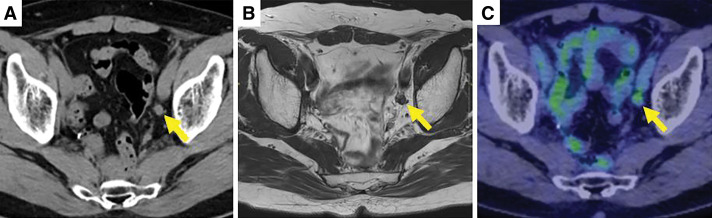
Plain CT, MRI T2 weighted, and PET-CT images taken 4.5 years after the initial surgery. CT and MRI show a 9-mm enlarged lymph node (arrows) (**A**, **B**), and PET-CT shows accumulation of SUVmax 2.08 (arrow) (**C**).

Then, 4.5 years after surgery, CT, MRI, and PET-CT revealed lymph node enlargement of 9 mm with SUVmax = 2.88 in the left lateral region (**Fig. 1**). Laparoscopic left lateral lymph node dissection was performed with a diagnosis of recurrence, but because a long time had passed since radiation therapy, severe fibrosis made removal difficult. In this surgery, the ureteral vasculature was preserved, and no ureteral stenosis was observed postoperatively. Although lymph node-like structures remained, there was little change in size on the images, and as there was no definitive diagnosis of recurrence, it was decided that the patient would be observed without treatment for the time being.

**Fig. 2:**
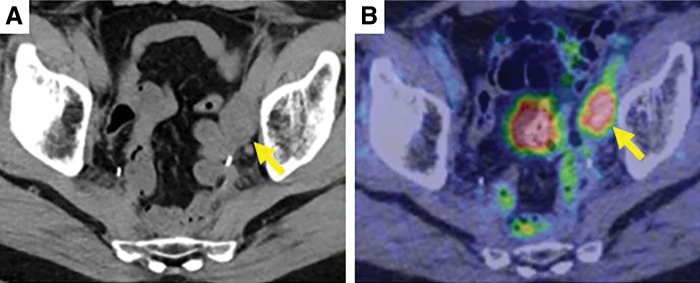
Plain CT and PET-CT images taken 7 years after the initial surgery. CT scan shows a 25-mm enlarged lymph node (arrow) (**A**), and PET-CT shows accumulation of SUVmax 8.06 (arrow) (**B**).

**Fig. 3:**
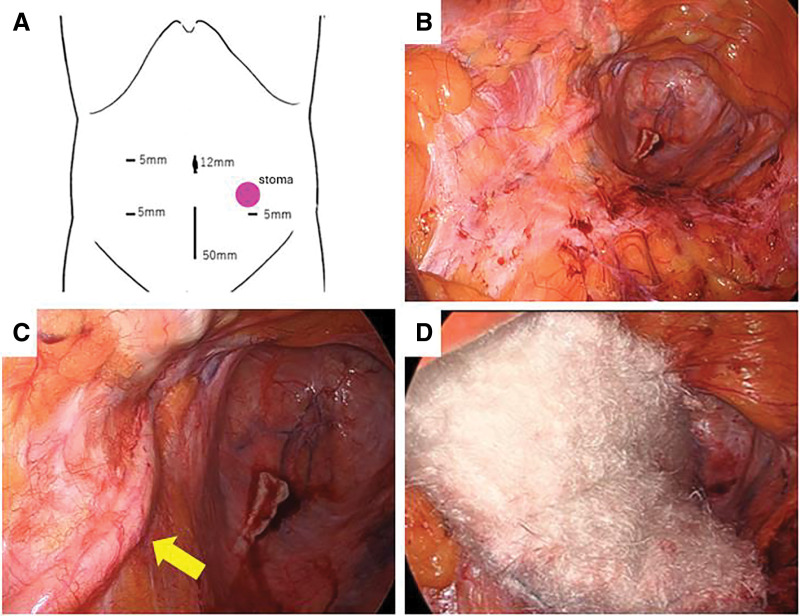
Intraoperative photograph of laparoscopic PGA spacer placement. The surgery was performed using 4 ports and a 5-cm small laparotomy (**A**). The adhesions at the promontory were removed (**B**). After lateral dissection, the ureter was exposed (arrow) (**C**). A PGA spacer was placed in the left pelvis (**D**). PGA, polyglycolic acid

Seven years after the initial surgery, CT and PET-CT showed that the lesion had clearly grown to 25 mm in size and had accumulated SUVmax = 8.06, strongly suggesting recurrence (**Fig. 2**). Reoperation was predicted to be difficult and the patient had already undergone CRT, so we decided to use CIRT and performed laparoscopic spacer placement before CIRT (Please see Endnotes section). A PGA spacer (15 mm wide, trimmed to 9 × 11 cm) was used. The surgery was performed using 4 ports, and a 5-cm small laparotomy was performed in the midline of the lower abdomen for spacer insertion (**Fig. 3A**). Only slight adhesions were observed around the promontory, which could be easily removed (**Fig. 3B**). No adhesions were observed around the left ureter, and the ureter was exposed (**Fig. 3C**). The spacer was placed at the planned placement site and fixed to the parietal peritoneum with absorbable sutures, taking care to prevent other organs from entering the radiation field (**Fig. 3D**). The right cranial and right caudal ends of the spacer were fixed to the dorsal peritoneum with a continuous suture using absorbable sutures, and the left cranial end was fixed to the peritoneum of the lateral wall with a continuous suture. Finally, the left caudal end was fixed to the peritoneum of the lateral wall with an interrupted suture while pulling the bladder to the right to prevent it from slipping under the spacer. An anti-adhesion sheet was applied over the spacer. The operation time was 150 min and blood loss was 15 mL.

**Fig. 4:**
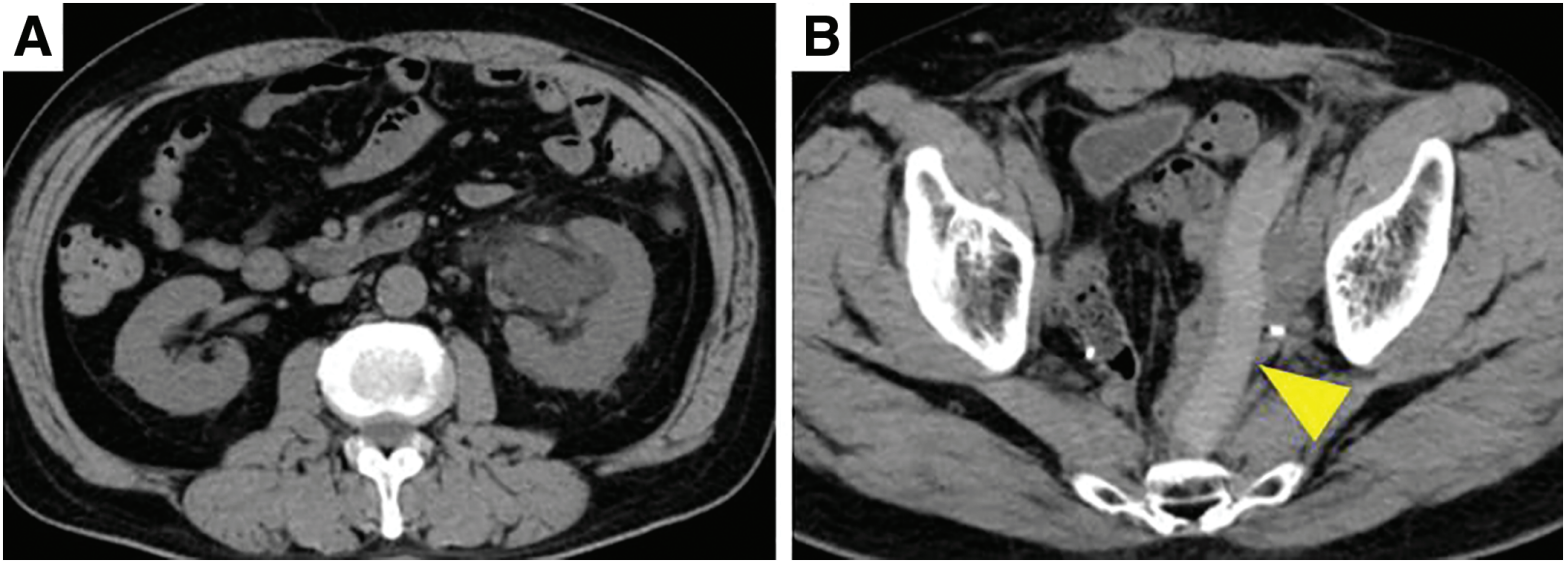
Plain CT image 7 days after PGA spacer placement. Left hydronephrosis was observed (**A**), and the cause was thought to be ureteral compression by the PGA spacer (arrowhead) (**B**). PGA, polyglycolic acid

**Fig. 5:**
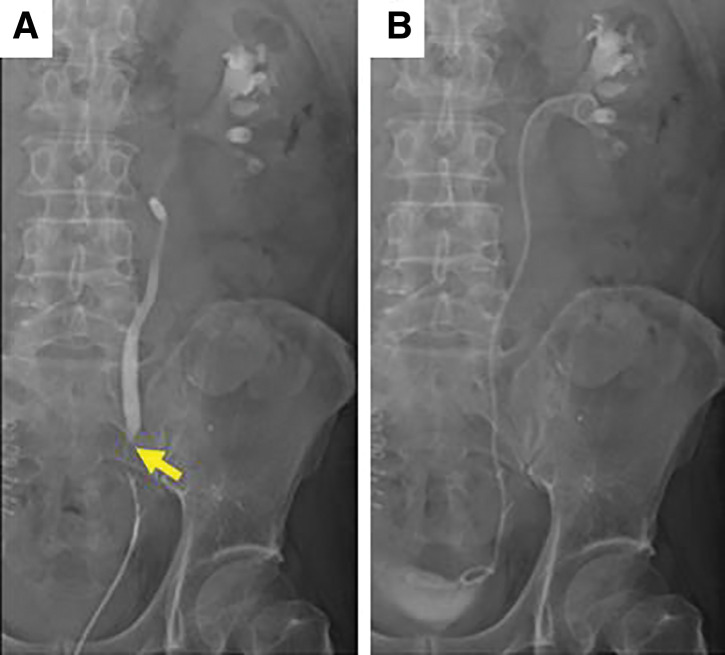
Ureterography image after PGA spacer placement. Ureteral stenosis was identified (arrow) (**A**), and a ureteral stent was placed (**B**). PGA, polyglycolic acid

**Fig. 6:**
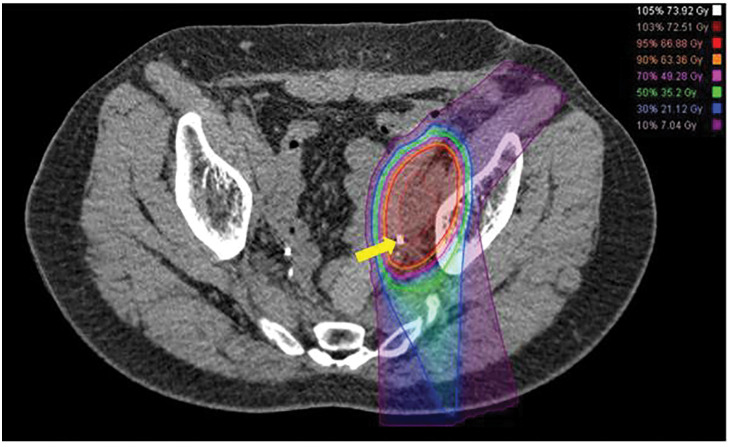
Dose distribution map of CIRT. The left ureter was very close to the target lymph nodes (arrow). CIRT, carbon-ion radiotherapy

**Fig. 7:**
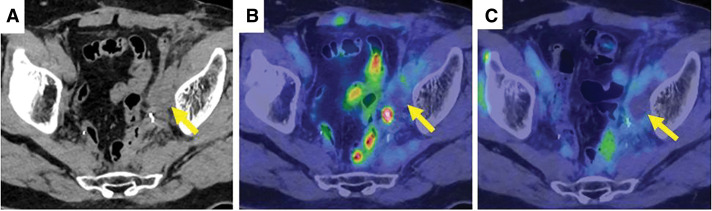
PET-CT 6 months and 18 months after CIRT. PET-CT 6 months after CIRT showed a shrinking trend and the accumulation had disappeared (arrows) (**A**, **B**). PET-CT 18 months after CIRT also showed no accumulation (arrow) (**C**). CIRT, carbon-ion radiotherapy

On POD 3, CT to investigate abdominal distension revealed left ureteral stenosis caused by compression by the spacer (**Fig. 4**). Follow-up CT was performed on POD 7, but the hydronephrosis had not improved, so the patient was discharged after placement of a left ureteral stent (**Fig. 5**). CIRT was started 1 month after the spacer placement, and irradiation was performed at 70.4 Gy/16 fr (**Fig. 6**). One month after the end of CIRT, we attempted to remove the ureteral stent and performed ureterography, but the stenosis was so severe that the ureteral stent had to be reinserted. At this time, the guidewire did not pass through the stricture when retrograde from the urethra, and the ureteral stent could be placed after puncturing the kidney and inserting the guidewire antegrade. Since then, the ureteral stent has been replaced regularly once every 3 months. Side effects from CIRT included swelling of the left lower limb. Regarding recurrent lesions, PET-CT 6 months after CIRT showed a reduction in size and no accumulation (**Fig. 7A**, **7B**). PET-CT 1.5 years after CIRT also showed no accumulation (**Fig. 7C**), meaning there was no local recurrence, and no distant metastasis had occurred.

## DISCUSSION

CIRT has attracted attention for its high local control effect against pelvic recurrence of rectal cancer.^3)^ In Japan, CIRT for pelvic recurrent rectal cancer became supported by health insurance in April 2022, and it is expected that the number of cases with utilization of CIRT will increase.

CIRT can be problematic, however, because irradiation of the adjacent gastrointestinal tract can lead to perforation and ulcer formation.^5)^ Aiming to solve this problem, a spacer is often inserted before CIRT to ensure a physical distance between the digestive tract and the area to be irradiated.^6)^ Bio-absorbable PGA spacers have recently attracted attention.^7)^ They retain their thickness even after insertion, maintaining 80% of their thickness for at least 8 weeks and then disappear within 1 year.^7,10,11)^ Conversely, non-absorbable spacers such as expanded polytetrafluoroethylene (ePTFE) sheets become foreign to the human body after CIRT, and reoperation may be required to remove them due to complications such as infection. Bio-absorbable spacers are thought to reduce this possibility. PGA spacer insertion is not widely reported, however,^7–9,11,12)^ and there is only one report of laparoscopic insertion.^13)^ The advantage of using a laparoscope when inserting a PGA spacer in this case is that it provides a good view when fixing it to the pelvic floor. There are few reported complications from PGA insertion, and while bowel obstruction and infection have been the most common, there have been no previous reports of hydronephrosis.^7–9,11)^ Furthermore, there have been no reports of ureteral stenosis occurring with other spacer materials.

In our case, hydronephrosis due to ureteral stenosis occurred soon after laparoscopic placement of a PGA spacer. The ureteral stenosis in the early postoperative period was thought to be caused by physical compression by the PGA spacer. In cases after lateral lymph node dissection, the ureter is exposed, meaning the PGA spacer can come into direct contact with it, which may increase the possibility of ureteral stenosis. The spacer was 0.2 g/cm^3^, weighing approximately 30 g in this case. In addition to the weight of the spacer, the additional pressure from the intestines that had fallen into the pelvic floor above the spacer was also thought to be the cause. Other possible explanations include the effects of previous radiation therapy, operations, and the severe inflammation induced by the spacer. Conversely, the persistence of ureteral stenosis even after the disappearance of the PGA spacer was thought to be a late adverse event of CIRT. In a report by Takiyama et al., the incidence of late adverse events of genitourinary reaction after CIRT for reirradiation cases was approximately 3.6%,^3)^ and there is a report of ureteral obstruction after CIRT in cases of upper tract ureteral cancer.^14)^ There have been some reports of ureteral stenosis as a complication of other radiation therapy.^15,16)^ In CIRT, it would be preferable to exclude the ureter from the radiation field if possible, but this was considered difficult in this case. If the stenosis is severe, placement of a ureteral stent may be difficult, which may lead to the need for nephrostomy. Therefore, prophylactic ureteral stenting before inserting a PGA spacer may be considered in cases of ureteral exposure after lateral dissection.

## CONCLUSIONS

Our patient had hydronephrosis due to ureteral stenosis, which occurred early after laparoscopic PGA spacer insertion. Although the ureteral stenosis in the early postoperative period was thought to be caused by the PGA spacer, the persistence of ureteral stenosis was thought to be a late adverse event of CIRT. In cases of ureteral exposure after lateral dissection with neoadjuvant chemoradiotherapy, prophylactic ureteral stenting before inserting a PGA spacer may be considered.

## ENDNOTES

The polyglycolic acid (PGA) spacer was inserted under the supervision of Hirotoshi Takiyama at QST Hospital. Carbon-ion radiotherapy (CIRT) was also performed at QST Hospital.

## DECLARATIONS

### Availability of data and materials

Data sharing is not applicable to this article, as no datasets were generated or analyzed.
